# Supplementary material for: Impact of midwife continuity of carer on stillbirth rate and first feed in England
Source: Commun Med (Lond). 2025 Aug 7;5:339. doi: 10.1038/s43856-025-01025-z (PMC12332168; doi:10.1038/s43856-025-01025-z)
Supplement: Supplementary file 1 — Supplementary Information [file 43856_2025_1025_MOESM1_ESM.pdf]

Supplementary information for impact of midwife continuity of carer on stillbirth rate and first feed in England

Chris Roebuck,[1,2,*] Jane Sandall,[3] Robert West,[4] Charlotte Atherden,[2] Kate Parkyn,[2] Owen Johnson,[1,*]

[1] School of Computing, **University of Leeds**, Leeds, United Kingdom, LS2 9JT.

[2] **NHS England**, 7 & 8 Wellington Place, Leeds, United Kingdom, LS1 4AP.

[3] School of Life Course and Population Sciences, Faculty of Life Sciences & Medicine, **King's College London**, 10th Floor, North Wing, St. Thomas' Hospital, London, United Kingdom, SE1 7EH.

[4] Leeds Institute of Health Sciences, **University of Leeds,** Worsley Building, Leeds, United Kingdom, LS2 9TJ.

[*] Chris Roebuck (Chris.Roebuck@nhs.net) and Owen Johnson (O.A.Johnson@leeds.ac.uk) are corresponding authors.

Supplementary Figure 1: Maternity Services Dataset (MSDS) data flow

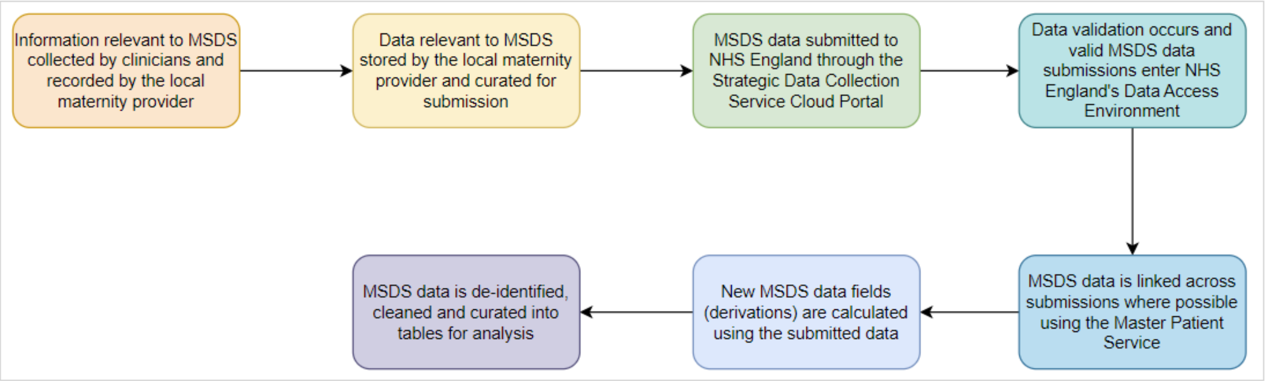

Stages in collecting and deriving the maternity services dataset from care being delivered in NHS Trusts to a curated data asset in NHS England.

Supplementary Figure 2: Cohort creation flow diagram

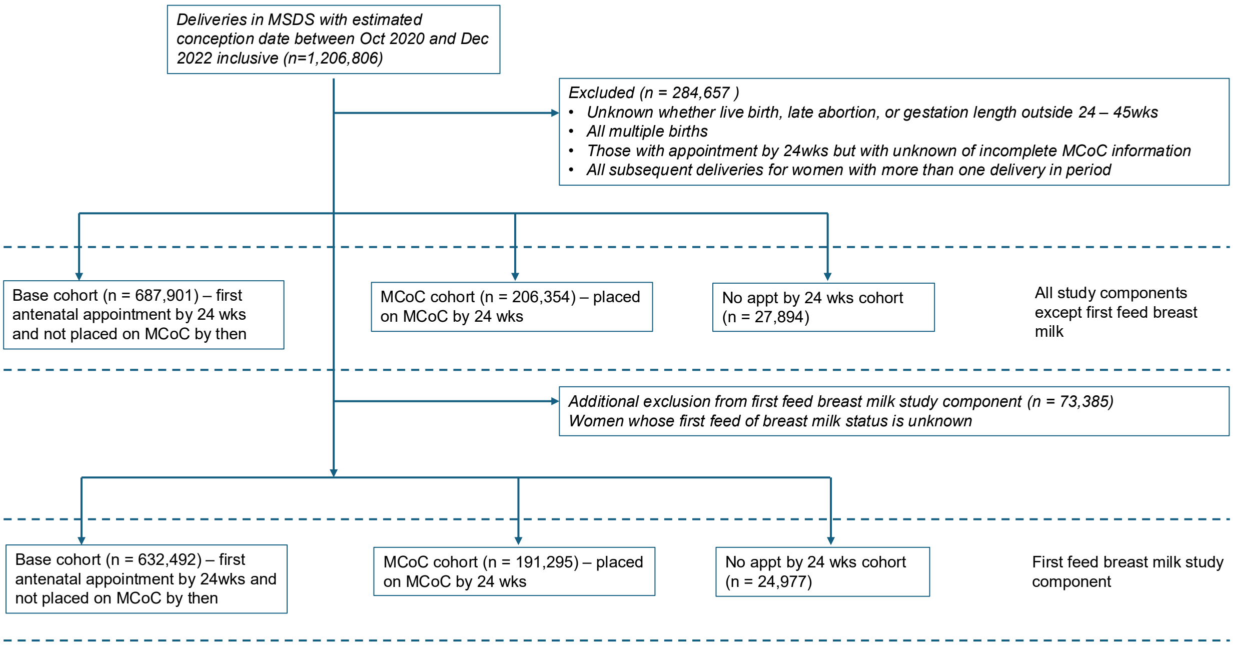

Incomplete MCoC information comprises: i) unknown MCoC status or ii) for those reported as being on MCoC but with no named midwife and/or care team.

After a series of exclusions applied sequentially, 922,149 women were included in the study looking at stillbirths and 848,764 women were included in the aspect looking at first feed of breast milk, once unknown outcomes removed. This comprised three distinct cohorts of women, the largest being women not placed on the MCoC pathway at 24 weeks (687,901 for stillbirth analysis; and 632,492 for first feed analysis), followed by women placed on the full MCoC pathway (206,354 for stillbirth analysis; and 191,295 for first feed analysis), with a separate smaller group of women who had no antenatal appointment by 24 weeks (27,894 for stillbirth analysis; and 24,977 for first feed analysis).

Supplementary Table 1: Summary of approach to missing data by item

Description of missing data item	Number of records	% of records in study	Approach	Rationale for approach
MCoC placement status unknown covering - i) women had appointment by 24 weeks but unknown whether placed on MCoC and ii) those women highlighted as being placed on MCoC but that did not meet all criteria including named midwife and care team.	224,047	n/a	Not included in study as per figure S1.	This variable underpinned the key study hypothesis, and while it could be assumed that women without a positive flag were very unlikely to have been placed on MCoC, this would not be certain, so only women positively confirmed as having been placed or not having been placed were included.
Outcome - unknown first feed of breast milk.	73,385	8.0%	Removed only from aspect of study looking at first feed breast milk.	Valid records for other aspects of study. For first feed of breast milk could only look at cases with known outcome.
Independent variable - ethnicity unknown.	24,221	2.6%	Gave each of these variables a distinct category of unknown that comprised these records and included them in regression models. Separately, performed sensitivity analysis of applying full case analysis (removing records with missing data for any of these variables), and imputation for missing data in these variables.	Decision not clear cut - full case analysis would exclude and potentially bias results; imputation reliant on assumptions, and other studies have created distinct categories for missing data. Most importantly, the sensitivity analysis shows the key study findings are the same regardless of the approach that was taken.
Independent variable - parity unknown.	18,730	2.0%		
Independent variable - previous caesarean unknown.	34,870	3.8%		
Independent variable - previous stillbirth unknown.	16,238	1.8%		
Independent variable - no English address provided.	6,378	0.7%	Included in study as distinct category.	Women in this category could be homeless or very recent immigrants so not have an address rather than this being a data entry issue and it is very important to include these women and represent them as a distinct group. This view is supported by the fact they had much higher rates of stillbirth than women with a recorded address.

Supplementary Note 1: Technical Specifications

Our logistic univariable and multivariable regression models were run on R version 4.2.1 using finalfit (1.0.7) and the default solver and other hyperparameters were applied. The multilevel models used the same packages but finalfit utilised the lmer function lme4 (1.1-35.1). Again, default hyperparameters were employed, except set nAGQ to 0. This is because, due to the volume of data, the system would not perform with any higher value of nAGQ. We explored running log binomial models for more intuitive outputs, but they did not converge when combined with multilevel modelling, so we used logistic regression models throughout.

The first part of the data pipeline was built on NHS England's data access environment using version 3.68 of Databricks. Within Databricks, we created a cleaned and curated set of definitive variables for each pregnancy using SQL alongside version 1.21.2 of Pandas within Python.

The statistical modelling on these variables to produce the final outputs was performed in R-studio (R version 4.2.1) on NHS England's remote desktop services. It employed the following R packages: Matrix (1.6-5), lme4 (1.1-35.1), dplyr (1.1.4), finalfit (1.0.7), openxlsx (4.2.5.2), DescTools (0.99.54), mlmhelpr (0.1.0), pROC (1.18.5).

A parallel run of some of the statistical modelling was done using Scikit-learn within Python to check it generated similar numbers to the R output as an additional layer of quality assurance.

Supplementary Figure 3: Relationship at NHS Trust level between proportion of women placed on MCoC at 24 weeks and the average IMD decile of women's address

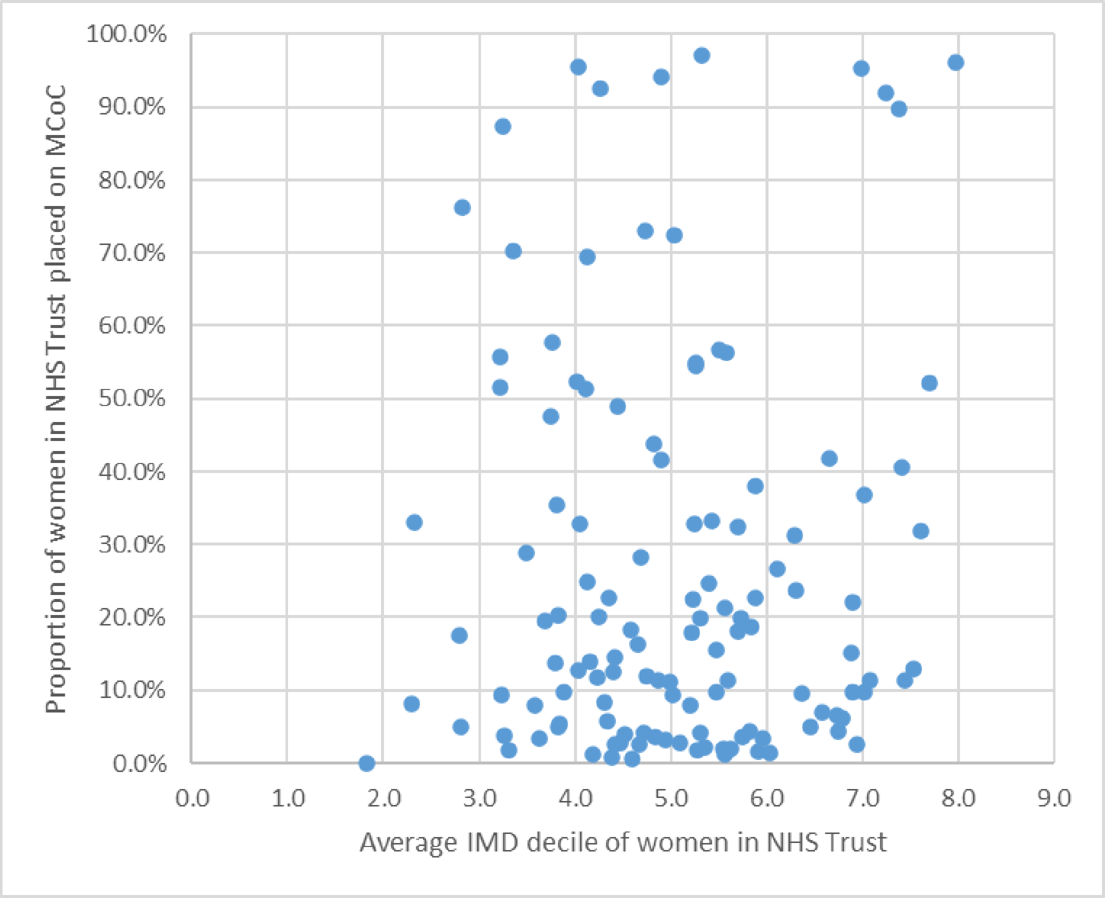

Average Index of Multiple Deprivation by NHS Trust obtained for all women in dataset. A higher average Index of Multiple Deprivation score relates to women living in less deprived areas. Only includes women with appointment and known MCoC placement status by 24 weeks. Covers English NHS Trusts conceptions from October 2020 – December 2022.

Supplementary Figure 3 shows no clear relationship at NHS Trust level between the mean Index of Multiple Deprivation of address of women included in the study at that Trust and the proportion of women placed on MCoC by 24 weeks.

Supplementary Table 2 shows that there is considerable geographical variation of the proportion of women with a known placement status who were placed on MCoC by 24 weeks. London and the South East regions had highest placement rates, both just over 30%, while the East of England had the lowest placement rate at 11.6%. The other four regions had placement rates between 17% and 25%.

Supplementary Table 2: Number and proportion of women placed on MCoC by 24 weeks by region of NHS Trust

Proportion only includes women with known MCoC placement status at 24 weeks; conceptions October 2020 – December 2022, England (numbers rounded to nearest 5).

NHS England Region	Proportion of women placed on MCoC	Number of women placed on MCoC
NHS England London	31.4%	47,525
NHS England South East	32.3%	43,180
NHS England South West	18.6%	13,705
NHS England Midlands	24.8%	43,700
NHS England East of England	11.6%	12,580
NHS England North East and Yorkshire	17.4%	25,720
NHS England North West	19.4%	19,940

Supplementary Figure 4 covers women and pregnancies included and excluded from the study. It shows a large change in reported MCoC placements over the course of 2020. Most women who were 24 weeks pregnant and conceived at the start of 2020 had unknown placement status reported, whilst those who conceived from October 2020 onwards, which is the point at which the study starts, had a known MCoC placement status by 24 weeks pregnancy. The numbers are broadly stable from this month onwards, with around 6,000-9,000 women with MCoC placements each month and 23,000 – 30,000 women known not to have been placed on MCoC per month. A smaller set of around 1,000 women per month had no antenatal appointment at all by 24 weeks pregnancy. In the study period, the number of women with an unknown MCoC status who are excluded peaks at just over 10,000 per month in October 2021 before coming down to just over 5,000 a month from May 2022.

Supplementary Figure 4: number of pregnancies by MCoC placement group at 24 weeks through time

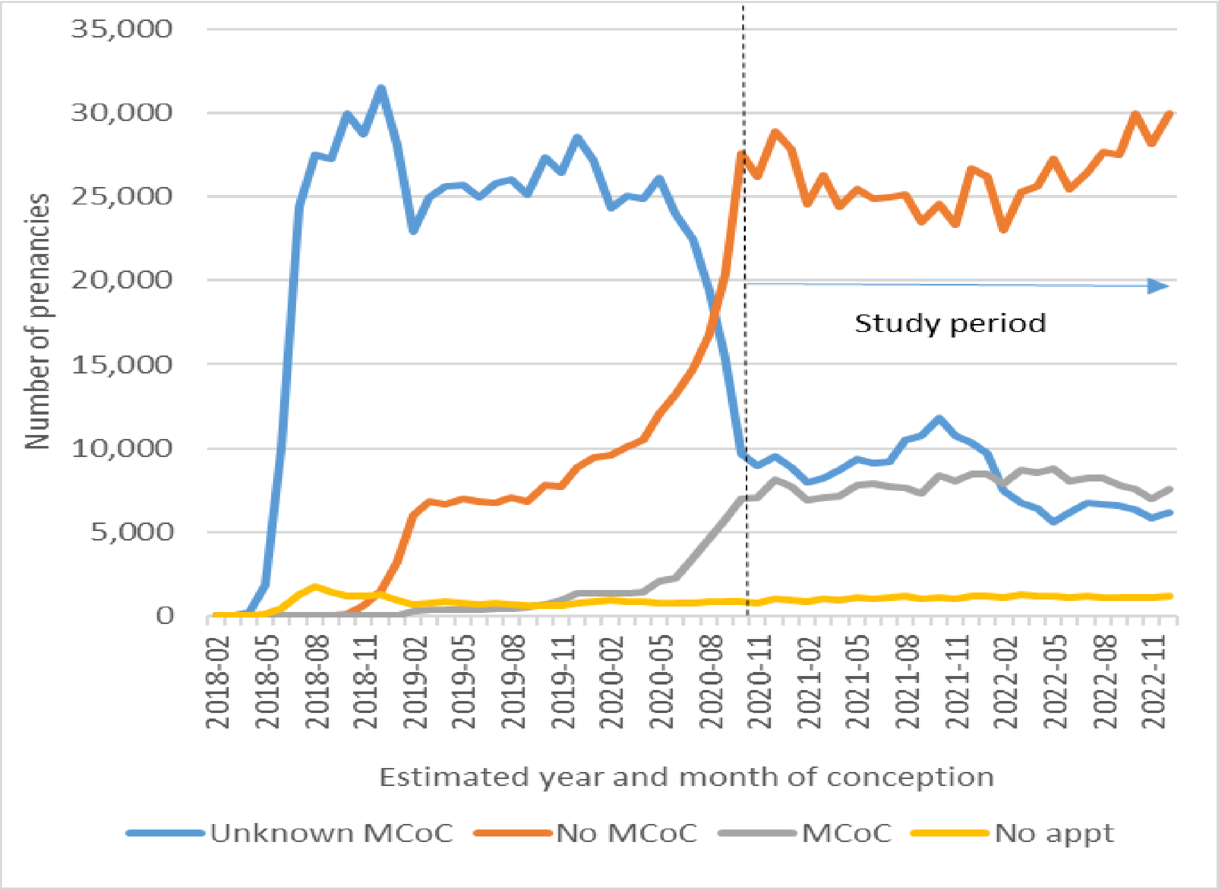

Blue line covers pregnancies for which the woman had an appointment by 24 weeks but it is unknown whether she was placed on MCoC at 24 weeks, including where incomplete information provided (note, this group is excluded from the study); dark orange line covers pregnancies for which it is confirmed that the woman was not placed on MCoC at 24 weeks; grey line covers pregnancies for which it is confirmed that the woman was placed on MCoC with named midwife and care team at 24 weeks; and yellow line covers pregnancies where woman did not have an appointment at all by 24 weeks.

Supplementary Table 3 shows some interaction between age and ethnicity, with women from Asian backgrounds having fewer than 1% of births aged under 20, whereas for White British women the figure is 3.0%.

Supplementary Table 3: Proportion of women in study in each age range for each ethnicity

	Under 20	20 to 24	25 to 29	30 to 34	35 to 39	40 to 44	45 and over	Total
White British (ref)	3.0%	13.2%	26.7%	34.9%	18.2%	3.8%	0.2%	100.0%
White Irish	2.2%	7.9%	16.1%	34.1%	30.9%	8.0%	0.8%	100.0%
Any other White background	2.3%	11.0%	22.7%	33.2%	24.1%	6.2%	0.5%	100.0%
White and Black Caribbean	4.7%	18.1%	27.6%	30.8%	15.0%	3.6%	0.2%	100.0%
White and Black African	3.0%	11.2%	27.2%	30.5%	21.8%	5.7%	0.6%	100.0%
White and Asian	2.4%	10.4%	23.2%	35.5%	22.8%	5.3%	0.5%	100.0%
Any other mixed background	3.0%	11.3%	23.1%	32.9%	23.4%	5.9%	0.5%	100.0%
Indian	0.2%	6.8%	25.4%	38.4%	23.9%	4.8%	0.5%	100.0%
Pakistani	0.6%	11.6%	31.3%	32.4%	18.9%	4.8%	0.3%	100.0%
Bangladeshi	0.5%	11.2%	32.4%	33.4%	18.1%	4.0%	0.4%	100.0%
Any other Asian background	0.7%	8.5%	24.4%	35.7%	23.8%	6.3%	0.6%	100.0%
Caribbean	2.5%	12.5%	24.0%	30.3%	22.6%	7.6%	0.5%	100.0%
African	0.7%	6.9%	27.8%	34.3%	22.5%	6.9%	1.0%	100.0%
Any other Black background	1.7%	9.2%	25.5%	32.4%	22.6%	7.6%	1.0%	100.0%
Chinese	0.1%	2.1%	11.7%	41.1%	34.8%	9.5%	0.6%	100.0%
Any other ethnic group	2.2%	11.6%	24.2%	32.2%	22.8%	6.4%	0.6%	100.0%
Unknown or not stated	2.0%	10.2%	25.1%	36.3%	20.9%	5.0%	0.4%	100.0%
Grand Total	2.4%	11.9%	26.1%	34.6%	20.0%	4.6%	0.4%	100.0%

Supplementary Table 4 shows a strong interaction between Index of Multiple Deprivation Decile of the woman's address and Ethnicity for women in the study. There was a relatively even distribution across deprivation deciles for women from a White British background. There was much variation in deprivation deciles between the individual South Asian groups with 31.6% of women of Pakistani ethnicity living in the most deprived decile and just 2.2% in the least deprived one, whereas for the Indian ethnicity 8.9% lived in the most deprived decile and 6.5% in the least deprived decile, with the third and fourth most deprived deciles being the most common for women from an Indian ethnicity with over 13% in each. Women of Black African ethnicity had 24.0% living in the most deprived decile, compared with 15.8% for those of Black Caribbean ethnicity.

Supplementary Table 4: Proportion of women in study living in each deprivation decile for each ethnicity

Deprivation deciles 1 = most deprived decile; 10 = least deprived decile												
	No English postcode	1	2	3	4	5	6	7	8	9	10	Total
White British	0.5%	11.6%	9.8%	9.3%	9.5%	9.9%	10.2%	10.2%	10.1%	10.0%	9.0%	100.0%
White Irish	0.8%	7.3%	9.1%	10.3%	10.6%	11.2%	10.3%	11.0%	10.1%	9.9%	9.5%	100.0%
Any other White background	0.8%	11.1%	12.8%	13.4%	12.1%	10.6%	9.9%	8.5%	7.9%	7.1%	5.9%	100.0%
White and Black Caribbean	0.5%	17.2%	16.4%	14.1%	10.6%	9.2%	8.1%	6.9%	5.9%	6.4%	4.7%	100.0%
White and Black African	0.7%	18.0%	15.5%	13.8%	12.3%	9.6%	7.2%	6.9%	6.4%	5.0%	4.7%	100.0%
White and Asian	0.5%	12.5%	10.6%	10.7%	10.6%	9.9%	9.4%	8.9%	9.0%	9.1%	8.8%	100.0%
Any other mixed background	0.9%	13.3%	14.4%	12.5%	11.9%	9.3%	9.3%	7.8%	7.5%	7.0%	6.1%	100.0%
Indian	2.1%	8.9%	12.4%	13.1%	13.5%	11.2%	10.0%	8.2%	7.3%	6.9%	6.5%	100.0%
Pakistani	0.6%	31.6%	19.3%	13.2%	10.4%	7.3%	5.2%	4.3%	3.4%	2.6%	2.2%	100.0%
Bangladeshi	0.6%	20.7%	23.7%	18.4%	11.5%	8.3%	5.6%	3.9%	3.0%	2.5%	1.8%	100.0%
Any other Asian background	1.3%	14.4%	13.3%	14.2%	12.2%	10.7%	9.1%	7.3%	6.7%	6.0%	4.7%	100.0%
Caribbean	0.5%	15.8%	21.6%	19.0%	12.6%	9.1%	7.0%	4.8%	3.9%	3.6%	2.1%	100.0%
African	1.4%	24.0%	19.6%	15.7%	10.9%	8.3%	6.2%	4.6%	3.9%	3.3%	2.1%	100.0%
Any other Black background	1.3%	15.9%	20.8%	16.9%	12.9%	9.6%	7.4%	5.4%	4.0%	3.6%	2.2%	100.0%
Chinese	0.8%	6.6%	9.5%	10.5%	10.2%	9.8%	10.6%	10.6%	10.0%	10.2%	11.2%	100.0%
Any other ethnic group	1.1%	18.2%	14.6%	14.5%	12.3%	9.3%	8.2%	6.9%	5.9%	5.2%	3.7%	100.0%
Unknown or not stated	1.1%	13.2%	11.5%	10.8%	10.6%	9.7%	9.5%	8.8%	8.8%	8.7%	7.2%	100.0%
Total	0.7%	13.4%	11.8%	11.1%	10.4%	9.8%	9.5%	8.9%	8.6%	8.3%	7.4%	100.0%

Supplementary Table 5 shows a strong interaction between the woman's age and deprivation of where they lived. There was a very skewed deprivation distribution for women under 20, with 28.4% of women living in the most deprived decile and 2.3% living in the least deprived decile. The distribution becomes progressively less skewed as age increases up to 30 to 34, where from this age band and higher, the distribution across deprivation deciles is quite even.

Supplementary Table 5: Proportion of women in study living in each deprivation decile for each age range

	Deprivation deciles 1 = most deprived decile; 10 = least deprived decile											
	No English postcode	1	2	3	4	5	6	7	8	9	10	Total
Under 20	0.8%	28.4%	18.1%	12.8%	9.7%	8.0%	6.6%	5.4%	4.3%	3.6%	2.3%	100.0%
20 to 24	0.9%	21.8%	16.1%	13.3%	10.9%	9.0%	7.7%	6.6%	5.7%	4.8%	3.3%	100.0%
25 to 29	0.8%	15.4%	13.3%	12.0%	10.8%	10.0%	9.3%	8.3%	7.8%	7.0%	5.4%	100.0%
30 to 34 (ref)	0.6%	10.6%	10.2%	10.2%	10.2%	10.0%	10.1%	9.8%	9.8%	9.6%	8.8%	100.0%
35 to 39	0.6%	9.6%	9.8%	10.1%	10.1%	9.9%	10.2%	9.8%	9.9%	10.0%	10.1%	100.0%
40 to 44	0.6%	10.4%	10.2%	10.4%	10.3%	9.8%	9.8%	9.6%	9.5%	9.7%	9.7%	100.0%
45 and over	0.5%	8.7%	11.0%	11.4%	9.5%	9.9%	9.9%	10.5%	8.9%	10.0%	9.7%	100.0%
Total	0.7%	13.4%	11.8%	11.1%	10.4%	9.8%	9.5%	8.9%	8.6%	8.3%	7.4%	100.0%

Supplementary Table 6 shows that the three different approaches to handling missing or unknown values for some variables (including them but as their own distinct category, assigning them to another category via imputation and removing records the include such values) do not alter the key findings of the study. Stillbirth rates for the full group of women placed on MCoC were slightly but not significantly lower than those not placed under all approaches. Also, under all approaches, stillbirth rates were much higher for women not seen at all by 24 weeks and still birth rates for Black women placed on MCoC were lower than Black women not placed with a quite large effect size and significant at $p<0.05$. Under all approaches, first feed of breast milk rates were significantly higher for women placed on MCoC than those not placed. The fact that key findings remained the same under all approaches was likely to be due to the relatively small volumes of missing and unknown data. While all three methods have closely aligned output, the two with the closest alignment are: including as their own category and imputation.

Supplementary Table 6: Sensitivity analysis of approaches to missing data. Comparison of model output for key findings under three different approaches to addressing missing and unknown values in the independent variables (Ethnicity, Parity, Previous Stillbirth, Previous Caesarean).

		OR (univariable)	OR (multivariable)	OR (multilevel)
Stillbirth rate - all demographics - MCoC	Study approach - separate category	0.943 (0.860-1.033, p=0.208)	0.936 (0.853-1.026, p=0.161)	0.983 (0.888-1.087, p=0.734)
	Impute - most common permutation	0.943 (0.860-1.033, p=0.208)	0.928 (0.846-1.017, p=0.112)	0.978 (0.884-1.082, p=0.664)
	Complete case analysis	0.950 (0.863-1.043, p=0.285)	0.937 (0.852-1.030, p=0.184)	0.979 (0.883-1.087, p=0.693)
Stillbirth rate - all demographics - +24 week first appointment	Study approach- separate category	2.391 (2.062-2.757, p<0.001)	2.136 (1.831-2.479, p<0.001)	2.161 (1.856-2.516, p<0.001)
	Impute - most common permutation	2.391 (2.062-2.757, p<0.001)	2.117 (1.816-2.456, p<0.001)	2.138 (1.837-2.488, p<0.001)
	Complete case analysis	2.092 (1.771-2.454, p<0.001)	1.831 (1.541-2.160, p<0.001)	1.849 (1.560-2.191, p<0.001)
Stillbirth rate - Black women - MCoC	Study approach - separate category	0.712 (0.515-0.964, p=0.033)	0.708 (0.511-0.962, p=0.032)	0.724 (0.526-0.995, p=0.047)
	Impute - most common permutation	0.712 (0.515-0.964, p=0.033)	0.717 (0.519-0.972, p=0.038)	0.727 (0.530-0.997, p=0.048)
	Complete case analysis	0.667 (0.470-0.924, p=0.019)	0.672 (0.473-0.931, p=0.021)	0.676 (0.482-0.950, p=0.024)
First feed breast milk - all demographics - MCoC	Study approach - separate category	1.156 (1.143-1.170, p<0.001)	1.157 (1.142-1.171, p<0.001)	1.072 (1.056-1.089, p<0.001)
	Impute - most common permutation	1.156 (1.143-1.170, p<0.001)	1.172 (1.157-1.186, p<0.001)	1.077 (1.060-1.093, p<0.001)
	Complete case analysis	1.103 (1.090-1.117, p<0.001)	1.138 (1.123-1.152, p<0.001)	1.070 (1.053-1.087, p<0.001)

OR (univariable) is odds ratio between target group and reference group for a specific demographic factor, with no other groups inputting to the logistic regression model. OR (multivariable) is odds ratio between target group and reference group for a specific demographic factor based on a single level logistic regression model with all demographic factors inputting. OR (level) is odds ratio between target group and reference group for a specific demographic factor based on a multilevel logistic regression model with all demographic factors inputting and NHS Trust as the random effects term. No English postcode leading to unknown value for Index of Multiple Deprivation was preserved as a separate category as this would cover recent immigrants and women of no fixed abode, who it is important to treat distinctly.

Supplementary Table 7 and figures Supplementary Figure 5 shows how well the different models predicted the different outcomes and whether including NHS Trust (through a multilevel model) as one of the adjustment factors helped improve prediction. They show that NHS Trust is a major factor in determining whether a woman is placed on MCoC and that it has some impact in determining whether a woman is not seen at all by 24 weeks. Including NHS Trust makes a very slight improvement to the ability to predict whether a woman has a first feed of breastmilk and has minimal impact on the ability to predict a stillbirth. Generally, the ability of the models to predict a stillbirth from the data available was very low, demonstrating there are many factors that determine stillbirth outside the available data.

Supplementary Figure 5 show very large variation between NHS Trust in MCoC placement and a large variation by NHS Trust in proportion of women not seen at all by 24 weeks from the models. They show much less variation between NHS Trust in stillbirth from the full study group from the models, with a much wider degree of uncertainty. Looking at stillbirth for Black women only the between Trust variation is even less and the uncertainty wider still.

Supplementary Table 7: summary of performance for all models, comparing for multivariate models, single level and models with random effects for provider.

	Single level (multivariate)			Multivariate with random intercept NHS Trust				
	AIC (lower = better but only comparable within row)	Log likelihood (higher = better but only comparable within row)	AUC (test data) Higher (above 0.5) = better model	AIC (lower = better but only comparable within row)	Log likelihood (higher = better but only comparable within row)	AUC (test data) Higher (above 0.5) = better model	Interclass correlation Higher = more impact of NHS Trust	Random effect standard deviation Higher = more impact of NHS Trust
First feed breastmilk, All Women	901,337	-450,625	0.71	878,626	-439,268	0.71	0.062	0.47
Stillbirths, All Women	38,330	-19,123	0.62	38,265	-19,089	0.62	0.02	0.26
Women placed on full continuity of carer pathway by 24 weeks (compared with women with first appointment by 24 weeks but known not to have been placed on pathway)	959,386	-479,651	0.55	670,793	-335,354	0.85	0.504	1.83
Women not seen at all by 24 weeks (compared with women with first appointment by 24 weeks but known not to have been placed on continuity of carer pathway)	210,950	-105,434		202,755	-101,336		0.171	0.82
Stillbirths, Black Women	3,523	-1,747	0.55	3,525	-1,746	0.52	0.007	0.16
Stillbirths, Women in most deprived population quintile based on postcode of usual address	11,925	-5,947		11,918	-5,943		0.013	0.21
Stillbirths, Women in least deprived 80% of population quintile based on postcode of usual address	25,921	-12,940		25,893	-12,924		0.016	0.23
Stillbirths, South Asian Women	6,825	-3,396		6,827	-3,396		0	0.00
Stillbirths, White Women	24,700	-12,335		24,636	-12,302		0.03	0.32
Stillbirths, Women of Mixed, Other and Unknown Ethnicity	3,292	-1,627		3,292	-1,626		0.02	0.26
First feed breastmilk, White Women	39,423	-19,682		38,344	-19,141		0.068	0.49
First feed breastmilk, Black Women	107,120	-53,529		104,572	-52,254		0.068	0.49
First feed breastmilk, South Asian Women	66,103	-33,018		63,672	-31,801		0.065	0.48
First feed breastmilk, Women of Mixed, Other and Unknown Ethnicity	684,781	-342,361		667,740	-333,839		0.067	0.49
First feed breastmilk, Women in most deprived population quintile based on postcode of usual address	252,660	-126,295		246,985	-123,457		0.06	0.46
First feed breastmilk, Women in least deprived 80% of population quintile based on postcode of usual address	639,983	-319,950		623,343	-311,630		0.064	0.47

Supplementary Figure 5: caterpillar plots showing ordered log odds ratios from models

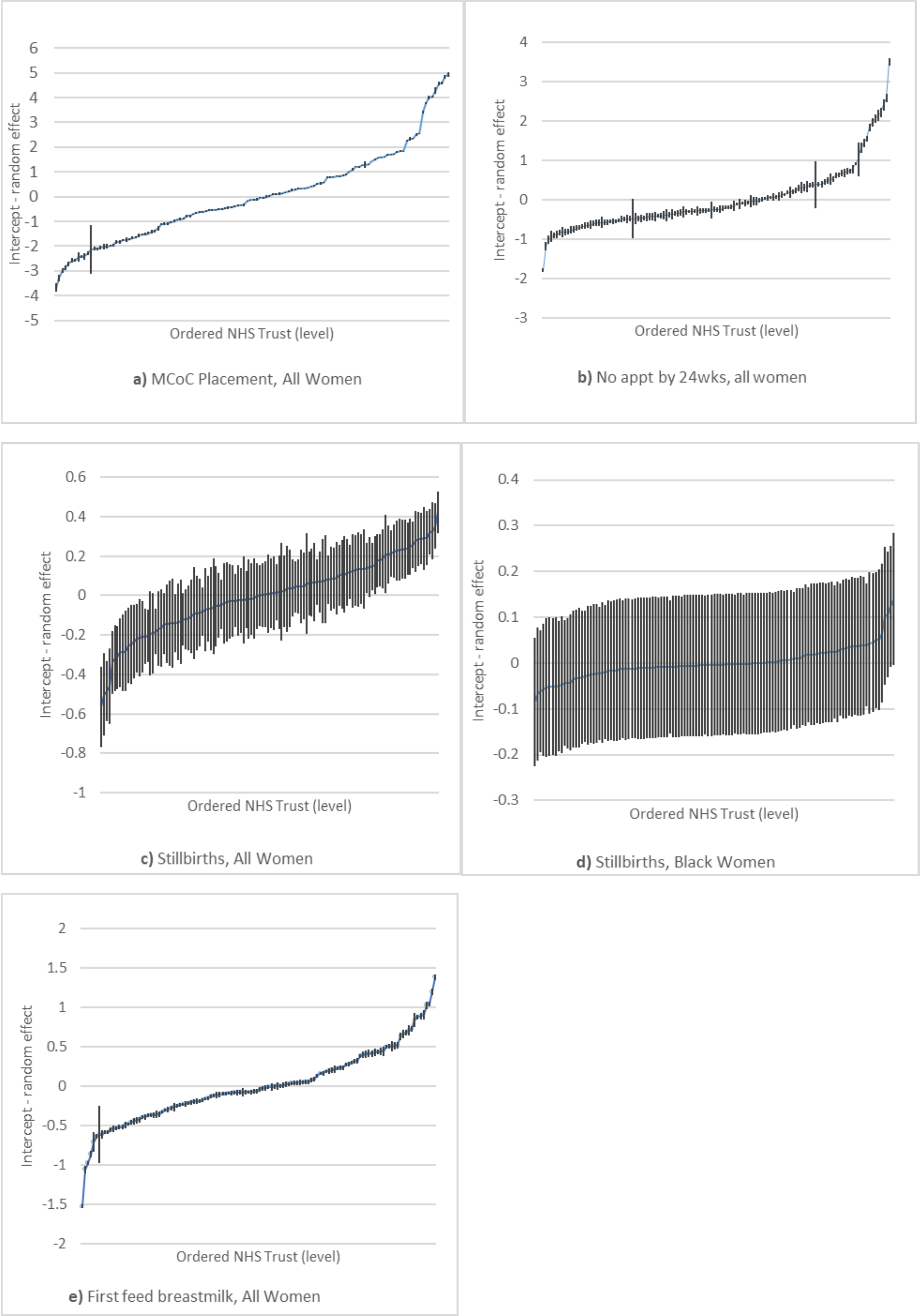

Ordered log odds ratios that generates random intercept for each NHS Trust in the multilevel models for with 95% confidence intervals. Each panel is labelled with the predicted outcome to which it relates.
