# Supplementary material for: Impact of midwife continuity of carer on stillbirth rate and first feed in England
Source: Commun Med (Lond). 2025 Aug 7;5:339. doi: 10.1038/s43856-025-01025-z (PMC12332168; doi:10.1038/s43856-025-01025-z)
Supplement: Supplementary file 2 — Description of Additional Supplementary Files [file 43856_2025_1025_MOESM2_ESM.pdf]

Description of Additional Supplementary Files

File name: Supplementary Data 1

Description: Summary of independent variables used in each model

File name: Supplementary Data 2

Description: Women in study who were placed on full continuity of carer pathway by 24 weeks as a proportion of those who had an appointment within 24 weeks with known continuity of carer status by group

File name: Supplementary Data 3

Description: Women in study who did not have a first antenatal appointment at all by 24 weeks as a proportion of those in study not placed on midwife led continuity of carer by 24 weeks by group.

File name: Supplementary Data 4

Description: Stillbirth rate for women in study by group

File name: Supplementary Data 5

Description: Still birth rate of women i) placed on midwife led continuity of carer pathway by 24 weeks, ii) having no appointment at all by 24 weeks compared with reference group of women who had an appointment by 24 weeks but were not placed on midwife led continuity of carer, for different demographic subgroups.

File name: Supplementary Data 6

Description: Rate of first feed of breast milk for women in study by group.

File name: Supplementary Data 7

Description: Rate of first feed of breast milk for women i) placed on midwife led continuity of carer by 24 weeks, ii) having now appointment at all by 24 weeks compared with reference group of women who had an appointment by 24 weeks but were not placed on midwife led continuity of carer, for different demographic subgroups.

File name: Supplementary Data 8

Description: Data underpinning Figure 1 and Supplementary Figure 3 – Distribution of MCoC placement and average IMD by NHS Trust

File name: Supplementary Data 9

Description: Data underpinning Supplementary Figure 4 - number of pregnancies by MCoC placement group at 24 weeks through time.

File name: Supplementary Data 10

Description: Data underpinning caterpillar plots in Supplementary Figure 5 showing ordered log odds ratios from models
