# Supplementary material for: Impact of midwife continuity of carer on stillbirth rate and first feed in England
Source: Commun Med (Lond). 2025 Aug 7;5:339. doi: 10.1038/s43856-025-01025-z (PMC12332168; doi:10.1038/s43856-025-01025-z)
Supplement: Supplementary file 13 — Reporting Summary [file 43856_2025_1025_MOESM13_ESM.pdf]

nature portfolio

Corresponding author(s): Chris Roebuck, Owen Johnson

Last updated by author(s): Jun 11, 2025

Reporting Summary

Nature Portfolio wishes to improve the reproducibility of the work that we publish. This form provides structure for consistency and transparency in reporting. For further information on Nature Portfolio policies, see our Editorial Policies and the Editorial Policy Checklist.

Statistics

For all statistical analyses, confirm that the following items are present in the figure legend, table legend, main text, or Methods section.

n/a	Confirmed	
☐	☒	The exact sample size (*n*) for each experimental group/condition, given as a discrete number and unit of measurement
☐	☒	A statement on whether measurements were taken from distinct samples or whether the same sample was measured repeatedly
☐	☒	The statistical test(s) used AND whether they are one- or two-sided<br>*Only common tests should be described solely by name; describe more complex techniques in the Methods section.*
☐	☒	A description of all covariates tested
☐	☒	A description of any assumptions or corrections, such as tests of normality and adjustment for multiple comparisons
☐	☒	A full description of the statistical parameters including central tendency (e.g. means) or other basic estimates (e.g. regression coefficient) AND variation (e.g. standard deviation) or associated estimates of uncertainty (e.g. confidence intervals)
☐	☒	For null hypothesis testing, the test statistic (e.g. *F*, *t*, *r*) with confidence intervals, effect sizes, degrees of freedom and *P* value noted<br>*Give P values as exact values whenever suitable.*
☒	☐	For Bayesian analysis, information on the choice of priors and Markov chain Monte Carlo settings
☒	☐	For hierarchical and complex designs, identification of the appropriate level for tests and full reporting of outcomes
☒	☐	Estimates of effect sizes (e.g. Cohen's *d*, Pearson's *r*), indicating how they were calculated

Our web collection on statistics for biologists contains articles on many of the points above.

Software and code

Policy information about availability of computer code

Data collection

No data were collected specifically for this study. The study re-used the Maternity Services Dataset (MSDS) which is used for many purposes locally and nationally in England. A description of the end-to-end data flow of MSDS is included in the supplementary information. Different local organisations would use different software and tools to capture their local data.

Data analysis

The code is available in an online repository.

The first part of the data pipeline was built on NHS England's data access environment using version 3.68 of Databricks. Within Databricks, we created a cleaned and curated set of definitive variables for each pregnancy using SQL alongside version 1.21.2 of Pandas within Python.
The statistical modelling on these variables to produce the final outputs was performed in R-studio (R version 4.2.1) on NHS England's remote desktop services. It employed the following R packages: Matrix (1.6-5), lme4 (1.1-35.1), dplyr (1.1.4), finalfit (1.0.7), openxlsx (4.2.5.2) ,DescTools (0.99.54), mlmhelpr (0.1.0) , pROC (1.18.5).
A parallel run of some of the statistical modelling was done using Scikit-learn within Python to check it generated similar numbers to the R output as an additional layer of quality assurance.

For manuscripts utilizing custom algorithms or software that are central to the research but not yet described in published literature, software must be made available to editors and reviewers. We strongly encourage code deposition in a community repository (e.g. GitHub). See the Nature Portfolio guidelines for submitting code & software for further information.

Data

Policy information about availability of data

All manuscripts must include a data availability statement. This statement should provide the following information, where applicable:

- Accession codes, unique identifiers, or web links for publicly available datasets
- A description of any restrictions on data availability
- For clinical datasets or third party data, please ensure that the statement adheres to our policy

All aggregate data supporting the findings of this study are available within the paper and its Supplementary Information. Data underpinning the figures and large tables in the article and its Supplementary Information are linked form this article as separate Excel files.
Other aggregated and anonymous Maternity Services Data relating to this study are available from the corresponding author on reasonable request.
The underlying record level data used in this research, including both the Maternity Services Dataset (MSDS) and the curated record level outcome file that fed the logistic regression models are de-identified but at patient record level, so a legal basis is required to obtain access. Access to the MSDS can be requested through NHS England's Data Access Request Service (DARS) https://digital.nhs.uk/services/data-access-request-service-dars and made available to requesters who meet the legal requirements. This is a chargeable service to cover the cost of running it.

Human research participants

Policy information about studies involving human research participants and Sex and Gender in Research.

Reporting on sex and gender	We did not collect any data specifically for this study, but reused a de-identified version of an existing dataset (Maternity Services Dataset) used in England for multiple purposes. Our study covers childbearing women. The Maternity Services Dataset does not currently capture information on gender identity so currently we are not able to report on this important area.<br>Our manuscript includes the following upfront statement: n this study, the term 'women' is used to refer to pregnant individuals as this terminology is used in the national level, secondary data source which was utilised in this study. The authors acknowledge that this term refers to all individuals who may be pregnant, such as transgender and nonbinary individuals, who may not identify as women.
Population characteristics	Detailed distributions of the study population of 922,149 people are included in the manuscript. Its coverage is all childbearing women who used the National Health Service in England and had their data recorded in the Maternity Services Dataset and whose estimated date of conception was between October 2020 and December 2022, subject to a few specific data exclusions set out in the Life Sciences study design section.
Recruitment	All women whose data were collected in the multi-purpose dataset and met the criteria set out in the population characteristics and Life Sciences section had their data incorporated in the study, so there was no specific recruitment for this study.
Ethics oversight	This study used existing data sources to evaluate the impact of MCoC. All data used in the study were from the de-identified Maternity Services Dataset (MSDS) for England, held by NHS Digital and NHS England. All data were analysed within the NHS Digital/NHS England secure data environments. Ethical approval was provided through the NHS Digital Request for Analysis Process on 9 September 2022. This approval required a senior requester for the analysis (the then Clinical Director for Maternity Services within NHS England) and review and approval of the use of data by the Information Asset Owner for the Maternity Services Dataset, and the Director responsible for Data Access within NHS Digital.

Note that full information on the approval of the study protocol must also be provided in the manuscript.

Field-specific reporting

Please select the one below that is the best fit for your research. If you are not sure, read the appropriate sections before making your selection.

☒ Life sciences ☐ Behavioural & social sciences ☐ Ecological, evolutionary & environmental sciences

For a reference copy of the document with all sections, see nature.com/documents/nr-reporting-summary-flat.pdf

Life sciences study design

All studies must disclose on these points even when the disclosure is negative.

Sample size	922,149 (all available data over time period rather than a sample). The study reused existing data rather than captured new data and used all available data for the time period for which uptake of Midwife Continuity of Carer reporting was sufficiently high, to maximise statistical power.
Data exclusions	Women whose pregnancy had unknown pregnancy outcome in maternity services dataset (could not be used in outcome analysis), late

Data exclusions	abortions (very different from stillbirths), or gestation length outside 24-to-45-week range (>45 weeks likely to be data quality issue; 24 weeks typically cut off for counting as still birth rather than miscarriage); all multiple births, those with appointment at 24 weeks who have unknown or incomplete information on Midwife Continuity of Care Status (could not clearly be placed into cohort), all but first delivery for women who have more than one delivery in the time period (including women multiple times may create bias). The number of women excluded under each of these categories is provided in table S1 of the supplementary information file. All exclusion criteria pre-established except unknown MCoC status and all but first delivery of women with multiple deliveries, which were based on expert advice that was received after the project started that women with unknown MCoC status could be driven by data quality rather than the fact they did not receive it; and that including women several times in the study could introduce bias.
Replication	The study used all available English data over the time period, rather than being based on a sample, so there are no data over the time period that have not been used in England that have not been used in the study so replication in England is not currently applicable.
Randomization	The study used comprehensive real-world data so the allocation of women to groups was based on real-world operational decisions across NHS hospitals in England rather than part of the study and was, therefore, not random. We looked to counteract this by standardizing by many variables as outlined in the study, to make as comparable as possible.
Blinding	This was not relevant given the re-use of real world data.

Reporting for specific materials, systems and methods

We require information from authors about some types of materials, experimental systems and methods used in many studies. Here, indicate whether each material, system or method listed is relevant to your study. If you are not sure if a list item applies to your research, read the appropriate section before selecting a response.

Materials & experimental systems

n/a	Involved in the study
☒	☐ Antibodies
☒	☐ Eukaryotic cell lines
☒	☐ Palaeontology and archaeology
☒	☐ Animals and other organisms
☐	☒ Clinical data
☒	☐ Dual use research of concern

Methods

n/a	Involved in the study
☒	☐ ChIP-seq
☒	☐ Flow cytometry
☒	☐ MRI-based neuroimaging

Clinical data

Policy information about clinical studies
All manuscripts should comply with the ICMJE guidelines for publication of clinical research and a completed CONSORT checklist must be included with all submissions.

Clinical trial registration	We have indicated that clinical data are involved in the study because the data used were originally sourced from clinical systems. This study does not fall within the ICMJE definition of a clinical trial because the research project did not prospectively assign people to interventions; instead the study reused existing data to evaluate the impact of different interventions for which the allocation of intervention to people had already been made for local operational reasons.
Study protocol	As above, this was not a clinical trial, but a reuse of already collected national data to evaluate the impact of a specific intervention that was rolled out via a national policy. Within NHS Digital, an internal request for analysis form was populated which provided the ethical approval from the Director with responsibility for analysis and Information Asset Owner to gain access to the de-identified dataset to proceed with the study.
Data collection	No data were collected specifically for this project, but it reused existing data that flowed from local maternity systems between October 2020 and March 2024.
Outcomes	We determined which outcomes to explore based on their importance and the quality of the data included in the Maternity Services Dataset, including understanding the volume and potential impact of missing data.
